# TAS2R supports odontoblastic differentiation of human dental pulp stem cells in the inflammatory microenvironment

**DOI:** 10.1186/s13287-022-03057-x

**Published:** 2022-07-28

**Authors:** Wen Kang, Yiwen Wang, Jiaying Li, Weige Xie, Dan Zhao, Li Wu, Hongwei Wang, Sijing Xie

**Affiliations:** 1 Department of Endodontics, Nanjing Stomatological Hospital, Medical School of Nanjing University, Nanjing, 210008 China; 2 State Key Laboratory of Analytical Chemistry for Life Science and Jiangsu Key Laboratory of Molecular Medicine, Medical School of Nanjing University, Nanjing, 210093 China

**Keywords:** Human dental pulp stem cells, Inflammation, Odontoblastic differentiation, TAS2R, TAS2R10

## Abstract

**Background:**

Inflammatory microenvironment promotes odontoblastic differentiation in human dental pulp stem cells (hDPSCs), but the regulatory mechanisms remain unclear. In this study, we aimed to explore the role of TAS2R in odontoblastic differentiation of hDPSCs in the inflammatory microenvironment.

**Methods:**

Microarray analysis was performed to explore the differential mRNA profiles in inflammatory and healthy pulp tissues from the patients. hDPSCs isolated from the healthy pulp tissues were stimulated by LPS, TNFα and IL-6, respectively, to verify the effect of TAS2R. The expression markers related to odontoblastic differentiation of hDPSCs were observed by qPCR and chemical staining methods. TAS2R10 was overexpressed or silenced to observe the effect on odontoblastic differentiation of hDPSCs under LPS stimulation. The G protein and intracellular Ca^2+^ were detected, respectively, by qPCR and Fluo-4AM Ca^2+^ fluorescent probe.

**Results:**

The expression of TAS2R was significantly upregulated in the inflammatory pulp tissues. In vitro, 5 subtypes of TAS2R mRNA expressions including TAS2R10, TAS2R14, TAS2R19, TAS2R30 and TAS2R31 in hDPSCs increased under the stimulation of LPS, TNFα or IL-6. In odontoblastic differentiation medium, we found LPS, TNFα or IL-6 stimulation promoted odontoblastic differentiation of hDPSCs. TAS2R10 overexpression in hDPSCs significantly increased the expression markers related to odontoblastic differentiation, whereas TAS2R10 silencing revealed the opposite effect. Furthermore, G protein was activated, and at the same time, intracellular Ca^2+^ enhanced when TAS2R10 was overexpressed, but decreased when TAS2R10 was silenced.

**Conclusions:**

This study demonstrated that TAS2R was found to be expressed in hDPSCs, and TAS2R promoted odontoblastic differentiation of hDPSCs by mediating the increase in intracellular Ca^2+^ via the G protein-coupled receptors (GPCR) conventional signaling pathway in inflammatory microenvironment, which may be a potential target for the development of effective conservative treatments for dental pulp repair.

## Background

Frequently, the inflammation and regeneration processes in dental pulp are often studied independently because they are antagonistic [1]. However, the emerging evidences indicate that inflammation is a prerequisite for pulp self-repair [2]. The dental pulp is well perceptive and resistant to inflammation, and has intricate defense mechanisms [3]. Low-level inflammation contributes to the pulp repair by migration of human dental pulp stem cells (hDPSCs) to the site of injury and eventual differentiation into restorative dentin [1, 4]. As the main cells of dental pulp repair, hDPSCs have the properties of mesenchymal stem cells with strong proliferative capacity, immunomodulatory properties and multidirectional differentiation potential [5]. hDPSCs even show remarkable neurogenic potential, since they originate from the neural crest [6, 7]. Many studies have found that inflammatory microenvironment can promote the odontoblastic differentiation of hDPSCs in vitro [8–10]. However, the mechanism of this process remains unclear. Recent studies have shown that bitter-taste receptors type 2 (TAS2R) which belongs to the G protein-coupled receptors (GPCR) family, plays an important role in the differentiation of stem cells. As Seo et al. [11] reported, TAS2R induces neuronal cells differentiation of cancer stem cells, thereby suppressing cancer stemness in human neuroblastoma cells. Wölfle et al. [12] reported that TAS2R promotes keratinocytes differentiation. Interestingly, in gingival fibroblasts of periodontitis [13] and gut epithelial cell of intestinal inflammation [14], the TAS2R expression is increased. Chemicals secreted by Gram-negative bacteria may be direct ligand of TAS2R, such as acyl-homoserine lactones (AHLs) [15]. Under LPS (a virulence factor released by Gram-negative bacteria) stimulation in vitro, the expression level of TAS2R is enhanced in lung macrophages [16]. Additionally, in the preliminary experiment, we observed that TAS2R was expressed in dental pulp, and the expression of TAS2R increased in inflammatory pulp tissues. We hypothesized that TAS2R might be expressed in hDPSCs and played a role in the differentiation of hDPSCs under inflammatory microenvironment.

In this study, we investigated the differential expression profiles between inflammatory and healthy pulp tissues by microarray and validated the results using qPCR. Then, the effect of TAS2R on promoting odontoblastic differentiation of hDPSCs under the inflammatory microenvironment in vitro was detected. This study might reveal the mechanism of pulpitis repair and might provide a new idea for conservative treatment of pulpitis.

## Materials and methods

### Collection of pulp samples

The inflammatory samples diagnosed as irreversible pulpitis according to the endodontic diagnosis system of the American Association of Endodontist [9] were obtained from the molar with deep caries and clinical symptoms were spontaneous pain, hot and cold irritation pain. The healthy samples were taken from non-carious impacted teeth. All samples used for study were taken from adults aged 18–28. Teeth with periodontitis were excluded. Samples were immediately frozen at −80 ℃ for subsequent experiments. Patients signed the informed consent. Approval was obtained from the Ethics Committee of Nanjing Stomatological Hospital, Medical school of Nanjing University.

### Microarray analysis

Total RNA was extracted from the pulp tissues by RNeasy Kit (QIAGEN, USA) according to the manufacturer’s instructions. Microarray analysis (OE Bio-tech, Shanghai, China) using inflammatory and healthy pulp tissues was performed. Differentially expressed RNAs were identified based on fold change ≥ 2.0 and *P* value ≤ 0.05. Afterwards, Gene ontology (GO) analysis and Kyoto Encyclopedia of Genes and Genomes (KEGG) analysis were applied to determine the roles of these differentially expressed mRNAs.

### Isolation, culture, flow cytometry identification and preconditioning of hDPSCs

hDPSCs were isolated from the third molars of healthy adults aged 18–28 years by enzymatic digestion method [17]. Briefly, the pulp tissues were extracted and cut into tiny pieces and digested with 5 mg/mL collagenase type P (Roche, Switzerland) for 30 min at 37 ℃ to obtain a single-cell suspension. The cells were cultured in Dulbecco’s modified Eagle’s medium (DMEM; Gibco, USA) supplemented with 10% fetal bovine serum (FBS; Gibco, USA), 100 U/mL penicillin, and 100 mg/mL streptomycin (Gibco, USA) at 37 °C and 5% CO_2_.

The third passage hDPSCs were identified via their surface antigen markers by flow cytometry. hDPSCs were resuspended and incubated in PBS (Gibco, USA) supplemented with 3% FBS for 45 min with primary antibodies against CD34, CD45, CD44 and CD146 [18]. Flow cytometer (BD FACSVerse, USA) was used for Flow cytometry analysis.

And the third passage hDPSCs were used for subsequent experiments. hDPSCs were stimulated with 1 µg/mL LPS [10], 10 ng/mL TNFα [19], 10 ng/mL IL-6 [20] for 12 h and 24 h, respectively, to produce inflammatory microenvironment.

### Odontoblastic differentiation induction of hDPSCs

The third passage hDPSCs (2 × 10^4^ cells/dish) were seeded in 35 mm culture dishes in odontoblastic differentiation medium (ODI) containing DMEM (Cat. No. 8122020; Gibco, USA) supplemented with 10% FBS, 1% Penicillin/Streptomycin, 50 µg/mL ascorbic acid (Sigma, USA), 10 mM β-glycerophosphate (Sigma, USA), 10 nM dexamethasone (Sigma, USA).

Quinine (QUN) as a bitter compound can effectively activate TAS2R10 [21]. 50 µM QUN (Sigma, USA) was added to the ODI 1 h before exposure to 1 µg/mL LPS (ODI + LPS + QUN). All medium were replaced every 2 days.

### siRNA and cell transfection

Small interfering RNA (siRNA) transfection was carried out using for TAS2R10 silencing studies. siRNA oligos were designed and synthesized by KeyGEN BioTECH Co. Ltd. (Nanjing, China). Non-specific siRNA-scramble were used as control. The sequences of siRNA-TAS2R10 were as follows:

Sense, 5′-GCUUUAUUCUCACCGGCUUTT-3′

Antisense, 5′-AAGCCGGUGAGAAUAAAGCCA-3′

The sequences of siRNA-scramble were as follows:

Sense, 5′-UUCUCCGAACGUGUCACGUdTdT-3′

Antisense, 5′-ACGUGACACGUUCGGAGAAdTdT-3′

hDPSCs were transferred to Opti-MEM medium (Gibco, USA). 50 nM siRNA and siRNA-scramble were transfected into hDPSCs via Lipofectamine 2000 (Thermo, USA) according to the manufacturer’s instructions. After 24 h of transfection, the medium was changed to odontoblastic differentiation medium supplemented with 1 µg/mL LPS (ODI + LPS + siRNA).

### ALP and Alizarin red staining

After 7 days or 14 days of odontoblastic differentiation, alkaline phosphatase (ALP) activity was evaluated by ALP staining [22]. The number of mineralized nodules was detected by Alizarin red staining [23]. hDPSCs were fixed with 4% paraformaldehyde for 30 min and washed with deionized water for 3 times at room temperature. hDPSCs were then stained with ALP (Beyotime, Shanghai, China) and Alizarin red (BestBio, Shanghai, China) using the corresponding assay kit according to the manufacturer’s instructions.

### Quantitative real-time PCR (qPCR)

hDPSCs were harvested to extract total RNA using RNA Isolation Kit (Vazyme, Nanjing, China). Reverse transcription was performed with PrimeScript RT Master Mix (TaKaRa, Japan) to synthesize first-strand cDNA. The mRNA expressions of ALP, runt-related transcription factor 2 (RUNX2), dentin sialophosphoprotein (DSPP), dentin matrix acidic phosphoprotein 1 (DMP-1), Gα and Gβ were measured by Viia 7 qPCR instrument (Applied Biosystems, USA) using SYBR Green Master Mix Reagent (Applied Biosystems, USA). Relative expression levels were determined by 2^−ΔΔCT^ method.

### Measurement of intracellular Ca^2+^

hDPSCs were cultured in black-walled, clear-bottom 96-well plates (Corning, USA) for 7 days. 2 µM Fluo-4 AM Ca^2+^ fluorescent probe (Beyotime, Shanghai, China) was added to each culture plate and incubated for 50 min at 37 °C with 5% CO_2_. Then, using Multi-Mode Microplate Reader (Spectramax M3, USA) to detect the intracellular Ca^2+^ fluorescence intensity.

### Statistical analysis

GraphPad Prism 7.0 software was used for One-way analysis of variance (ANOVA) and Student’s t-test. All experiments were repeated at least three times. The experimental data were expressed as mean ± standard deviation, and *P* < 0.05 was considered statistically significant.

## Results

### TAS2R was significantly upregulated in the inflammatory pulp tissues in mRNA profiles

Microarray technology could reveal the differential expression profiles between inflammatory and healthy pulp tissues, which might help us discover the genes or pathways that played a key role in inflammation promoting differentiation of hDPSCs. Thus, we collected a total of 4 pulp samples, including 2 inflammatory samples and 2 healthy samples for microarray analysis. Two inflammatory samples were obtained from the mandibular first molar. The average age was 25, including 1 male and 1 female. Two healthy samples were taken from the mandibular third molar. The average age was 22, including 1 male and 1 female. By extracting total RNA and measuring the RNA concentration, the purity, the quality of the 4 samples was tested and could be used for the subsequent experiments.

**Fig. 1:**
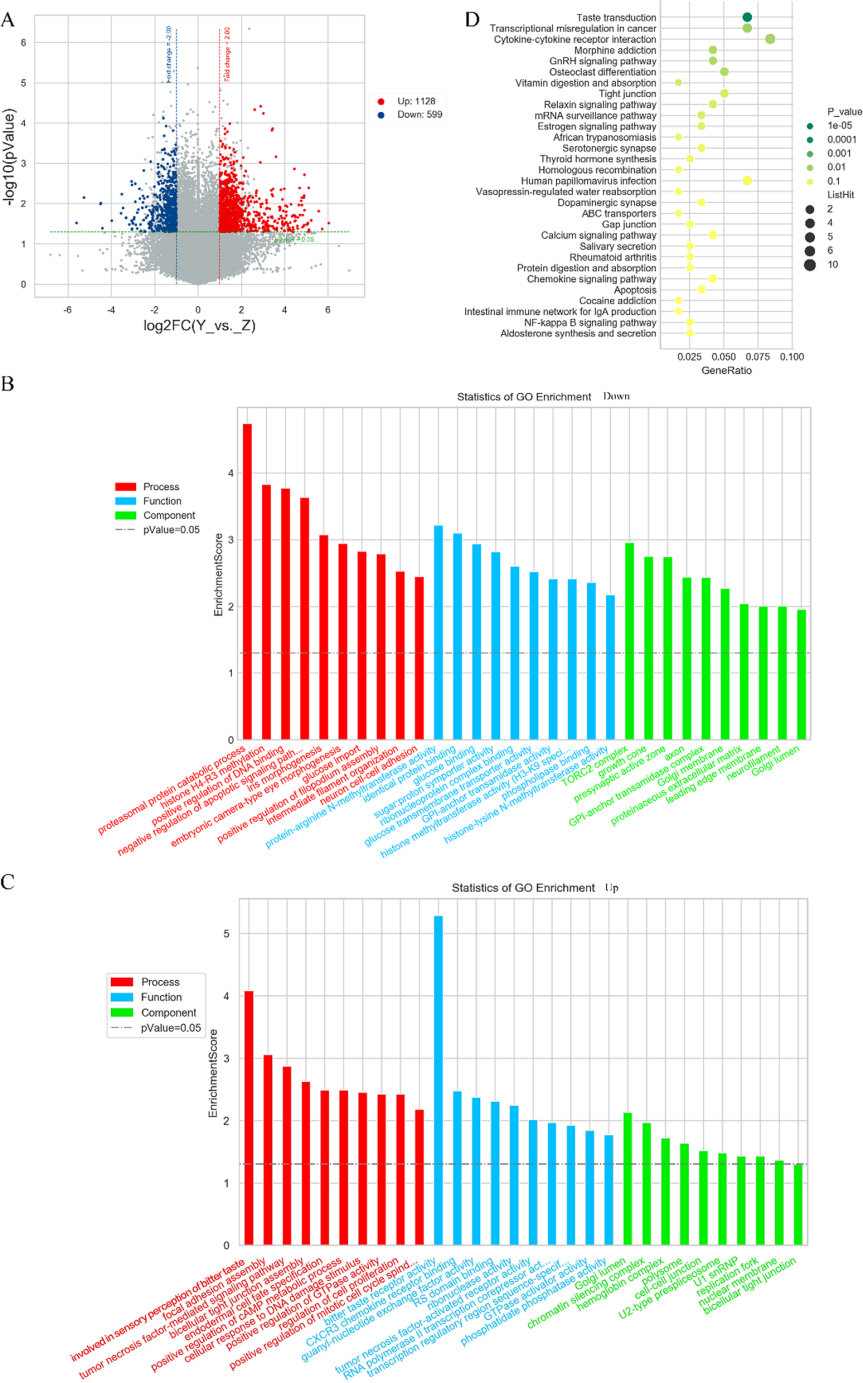
Differential mRNA expression profiles of inflammatory and healthy pulp tissues. **a** Volcano plots of mRNA differences. Axis represents log2-scaled fold changes and *p* value (− log10 scaled). The red and blue points represent up-and downregulated mRNAs. **b** The GO analysis of top 10 downregulated gene pathways in biological process, molecular function, cellular component, respectively. **c** The GO analysis of top 10 upregulated gene pathways in biological process, molecular function, cellular component, respectively. **d** Scatter plots of top 30 KEGG pathways. The vertical axis corresponds to pathways, the horizontal axis represents GeneRatio, the size of the dot corresponds to the number of different genes, the color of the dot represents the significance of the difference

According to the analysis of differential mRNA profiles in inflammatory and healthy pulp tissues, the volcano plots showed 1727 differential genes between the two groups, of which 1128 were upregulated and 599 were downregulated (Fig. 1a). By GO enrichment analysis, the differentially up- or downregulated gene pathways were enriched including biological process, molecular function, and cellular component, and the top 10 upregulated and top 10 downregulated GO items were showed. The gene pathways among the downregulated were mainly related to protein metabolism, histone methylation, positive regulation of DNA bonding, cell proliferation, apoptosis and other functions (Fig. 1b). We also found that upregulated gene pathways were mainly involved in regulating cell proliferation, growth, metabolism, adhesion and other functions. And the top 1 upregulated gene pathway was associated with TAS2R (Fig. 1c). The KEGG analysis was used to explore which pathways might be associated with the differential genes, and the scatter plots showed that the pathways with the most abundant genes and the most significant differences were related to TAS2R transduction (Fig. 1d). Genes enriched in TAS2R pathway are shown in Table 1.

**Table 1:** The differential genes of TAS2R pathway between inflammatory and healthy pulp tissues

Gene name	Fold change	*P*-value	Regulation
TAS2R10	3.67	0.039	Up
TAS2R14	3.17	0.001	Up
TAS2R19	2.04	0.030	Up
TAS2R30	2.91	0.041	Up
TAS2R31	2.25	0.045	Up

### TAS2R was increased in hDPSCs under LPS, TNFα or IL-6 stimulation

**Fig. 2:**
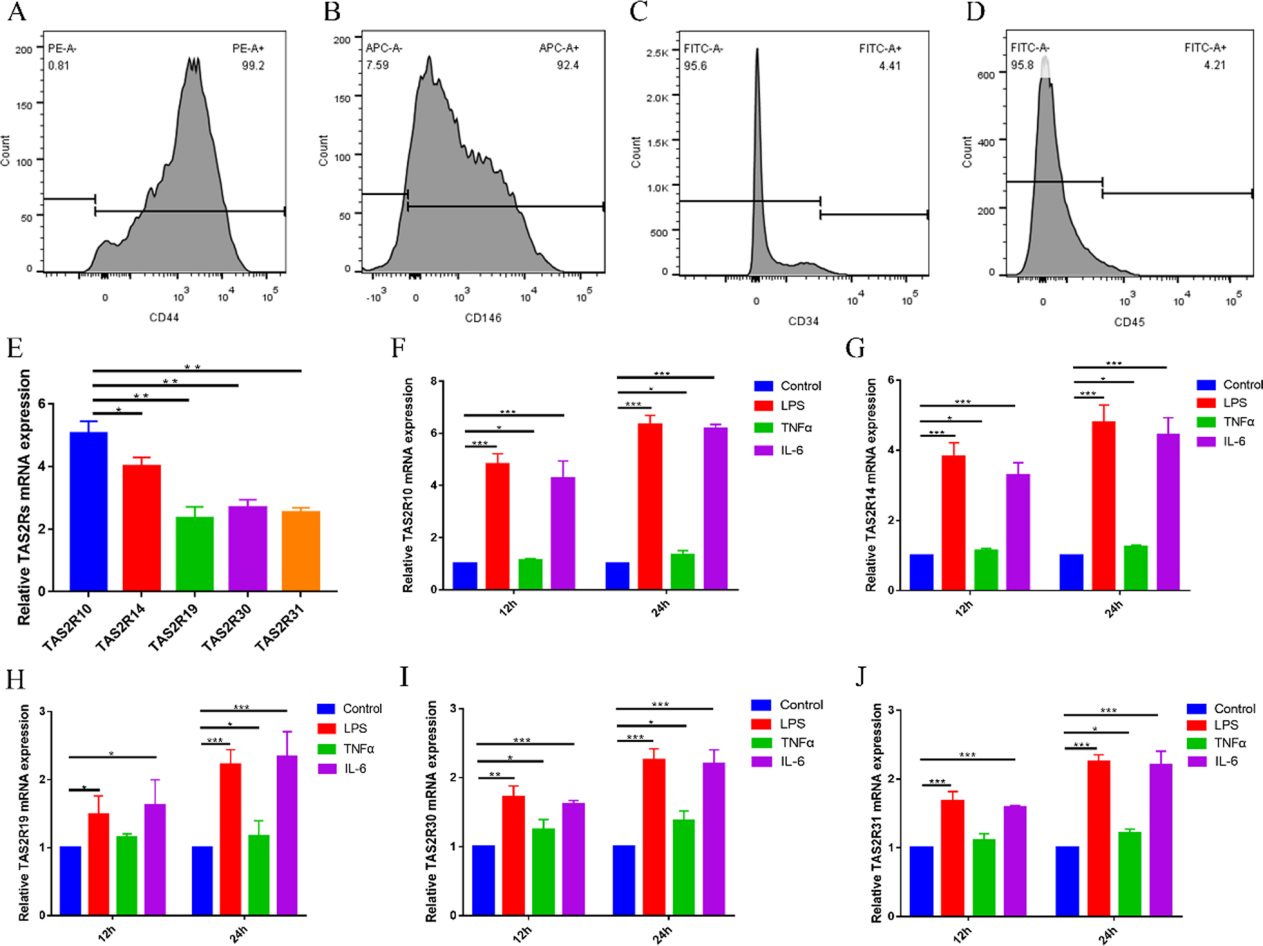
Flow cytometry results of the surface marker and the expression of TAS2R in hDPSCs stimulated by LPS, TNFα or IL-6. **a–d** hDPSCs were positive for CD44, CD146 and negative for CD34, CD45. **e** TAS2R10, TAS2R14, TAS2R19, TAS2R30 and TAS2R31 were found to be expressed in hDPSCs. **f–j** The mRNA expressions of TAS2R10, TAS2R14, TAS2R19, TAS2R30 and TAS2R31 in hDPSCs after 12 h and 24 h stimulation with 1 µg/mL LPS, 10 ng/mL TNFα and 10 ng/mL IL-6, respectively. *P < 0.05; **P < 0.01; ***P < 0.001

As hDPSCs are the main cells of dental pulp repair under injurious stimuli, we investigated the expression of TAS2R in hDPSCs. hDPSCs were cultured in vitro and the expression of TAS2R in hDPSCs was detected by qPCR. The results showed that hDPSCs were successfully isolated and cultured from the pulp of impacted teeth, and hDPSCs were positive for stem cell surface markers CD44, CD146 and negative for CD34, CD45 by flow cytometry analysis (Fig. 2a–d). qPCR results showed that there were mRNA expressions of TAS2R10, TAS2R14, TAS2R19, TAS2R30 and TAS2R31 in hDPSCs (Fig. 2e).

To verify the microarray results, we stimulated hDPSCs with LPS, TNFα and IL-6 to mimic an inflammatory microenvironment, and detected the expression of TAS2R in hDPSCs by qPCR. The dominant bacteria in pulpitis are Gram-negative anaerobic bacteria [24]. LPS, a virulence factor from Gram-negative bacteria, is released at high levels in pulpitis [25]. In addition, TNFα and IL-6 are also highly expressed in the pulpitis [26]. Therefore, LPS, TNFα and IL-6 were selected in our study to stimulate hDPSCs to produce an inflammatory microenvironment. The results showed that the mRNA expression of TAS2R10, TAS2R14, TAS2R19, TAS2R30 and TAS2R31 increased after stimulation of hDPSCs with 1 µg/mL LPS, 10 ng/mL TNFα and 10 ng/mL IL-6 for 12 h and 24 h, respectively (Fig. 2f–j). Since TAS2R10 expression was highest in hDPSCs and increased significantly under the inflammatory microenvironment. TAS2R10 was selected for the subsequent experiments.

### TAS2R was involved the odontoblastic differentiation of hDPSCs in the inflammatory microenvironment

**Fig. 3:**
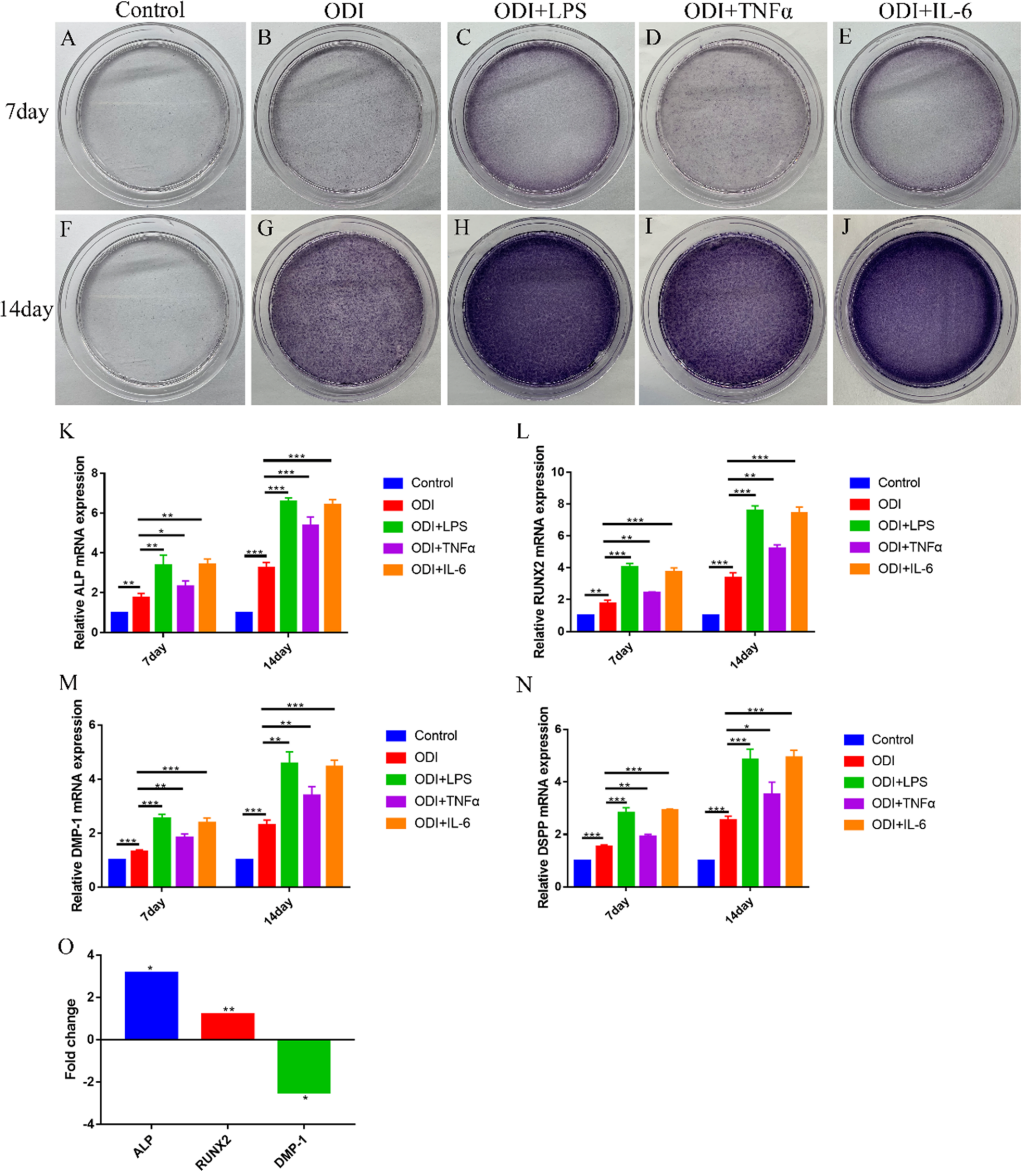
LPS, TNFα or IL-6 promoted odontoblastic differentiation of hDPSCs. **a–j** hDPSCs were cultured in ODI containing with 1 µg/ml LPS, 10 ng/mL TNFα and 10 ng/mL IL-6, respectively, for 7 days and 14 days and then stained with ALP. hDPSCs in DMEM medium were used as control. **k–n** The mRNA expressions of ALP, RUNX2, DMP-1, DSPP were analyzed by qPCR. **o** The mRNA expression fold changes of ALP, RUNX2, DMP-1 in microarray. *P < 0.05; **P < 0.01; ***P < 0.001

To demonstrate that the inflammatory microenvironment promoted differentiation of hDPSCs, hDPSCs were stimulated with 1 µg/mL LPS, 10 ng/mL TNFα or 10 ng/mL IL-6 in ODI for 7 days and 14 days, respectively. The results showed that LPS, TNFα and IL-6 could improve ALP activity (Fig. 3a–j) and enhance the expression markers related to odontoblastic differentiation including ALP, RUNX2, DMP-1 and DSPP (Fig. 3k–n). And 1 µg/mL LPS was selected to produce inflammatory microenvironment for subsequent experiments.

Meanwhile, the differential expressions of ALP, RUNX2, DSPP and DMP-1 were detected by microarray between inflammatory and healthy pulp tissues. The results showed that the mRNA expressions of ALP and RUNX2 were upregulated, but DMP-1 expression was downregulated in inflammatory pulp tissues. There was no difference in DSPP expression (*P* > 0.05) (Fig. 3o).

**Fig. 4:**
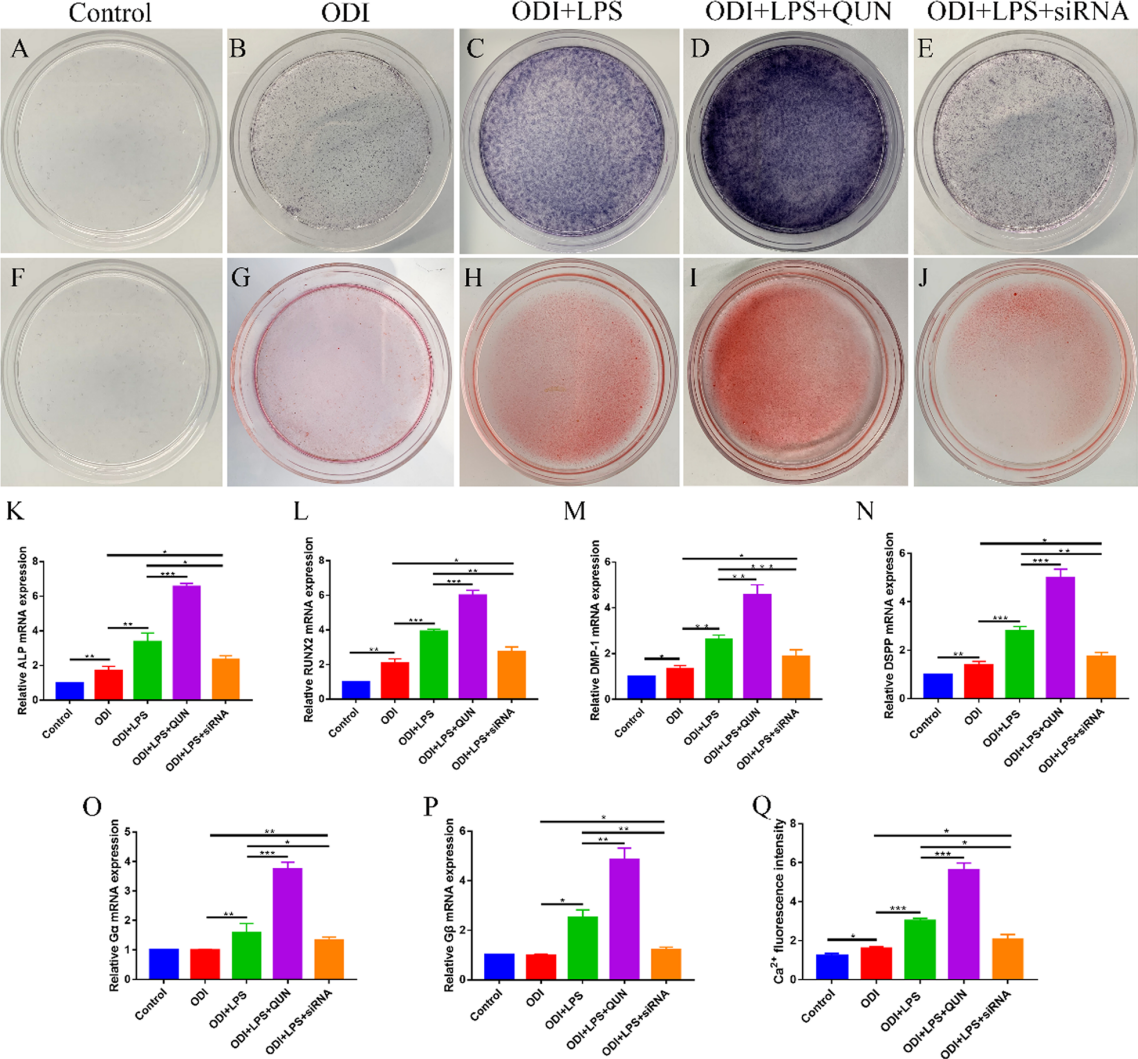
TAS2R was involved in the regulation of odontoblastic differentiation of hDPSCs and its mechanism. **a–e** The results of ALP staining at 7 days. **f-j** The results of Alizarin red staining at 7 days. **k–n** the mRNA expressions of ALP, RUNX2, DMP-1 and DSPP. **o, p** The mRNA expressions of Gα, Gβ. **q** Intracellular Ca^2+^ in hDPSCs. *P < 0.05; **P < 0.01; ***P < 0.001

In the above experiments, we found that inflammatory microenvironment (such as LPS-induced inflammatory microenvironment) could promote the odontoblastic differentiation of hDPSCs. And the TAS2R expression (such as TAS2R10) was increased under LPS stimulation. To examine whether the LPS-induced differentiation of hDPSCs was dependent on TAS2R10, hDPSCs were co-treated with LPS and the TAS2R10 agonist QUN (50 µM) or siRNA-TAS2R10 transfection. Results showed that TAS2R10 overexpression in hDPSCs significantly enhanced the ALP activity and mineralized nodules formation, whereas TAS2R10 silencing revealed the opposite effect (Fig. 4a–j). The results of qPCR demonstrated that the expressions of ALP, RUNX2, DMP-1 and DSPP were remarkably increased when TAS2R10 was overexpressed, but decreased when TAS2R10 was silenced (Fig. 4k–n). These data revealed that TAS2R10 played a positive regulatory role in odontoblastic differentiation of hDPSCs.

### TAS2R positively regulated intracellular Ca^2+^ and promoted the odontoblastic differentiation of hDPSCs under LPS-stimulation

Gα and Gβ are two subunits of G protein, which are downstream molecules of TAS2R.

The mRNA expressions of Gα and Gβ were detected by qPCR. The results showed that the expression levels of Gα and Gβ in ODI group were similar to the Control group, but increased under LPS stimulation, indicating that TAS2R10 was activated. The expressions of Gα and Gβ were the highest in ODI + LPS + QUN group, but decreased in ODI + LPS + siRNA group (Fig. 4o, p).

An increase in intracellular Ca^2+^ is a typical downstream effect mediated by TAS2R. Previous studies have reported that intracellular Ca^2+^ positively regulates the odontoblastic differentiation of hDPSCs [27, 28]. Therefore, Fluo-4 AM Ca^2+^ fluorescent probe was used to evaluate the intracellular Ca^2+^ in this study. The results showed that the intracellular Ca^2+^ was increased in the ODI group compared with the Control group. After LPS stimulation, the intracellular Ca^2+^ further enhanced. The intracellular Ca^2+^ were the highest in ODI + LPS + QUN group, but decreased in ODI + LPS + siRNA group (Fig. 4q). It was suggested that activation of TAS2R10 lead to an increase in intracellular Ca^2+^.

## Discussion

Dental caries is the most important factor that cause inflammation of dental pulp and may eventually lead to the pulp necrosis [29]. However, the emerging evidences indicate that inflammation is an essential stage in pulp tissue repair and regeneration [30]. When the dental pulp is inflamed, hDPSCs migrate to the site of injury and differentiate into odontoblasts, forming reparative dentin and protecting pulp vitality [31]. Therefore, the more thoroughly study on the mechanisms of odontoblastic differentiation of hDPSCs, the more reliable the conservative treatment of pulpitis will be. Huang et al. [9] found that inflammation-induced overexpression of microRNA-223-3p regulates odontoblastic differentiation of hDPSCs by targeting SMAD3. Xu et al. [19] reported that miRNA-21 positively regulates odontoblastic differentiation of hDPSCs coordinating with STAT3 in the inflammatory microenvironment.

In our study, we collected inflammatory and healthy pulp tissues for differential expression profiles analysis to explore the genes that were overexpressed or silenced in the inflammatory conditions. We found that TAS2R was significantly up-regulated in the inflammatory pulp tissues and LPS-stimulated hDPSCs. TAS2R was found to be expressed in hDPSCs for the first time. TAS2R was initially identified in taste buds of the tongue and played a role in recognizing bitter taste, preventing the ingestion of toxic substances and performing self-defense functions [32]. There is increasing evidence that TAS2R has a variety of functions independent of taste in anti-inflammation [16], anti-infection [14], reduction airway hyper-responsiveness [33]. Recent studies have reported that TAS2R plays an important role in the regulation of keratinocytes and cancer stem cells differentiation [11, 12]. Therefore, it was reasonable that TAS2R increased to regulate the process of odontoblastic differentiation in hDPSCs. Grassin-Delyle et al. [16] reported that TAS2R expression is increased in lung macrophages from patients with pneumonia and plays an anti-inflammatory role by inhibiting the production of inflammatory cytokines (TNF-α, CCL3, CXCL8 and IL-10). The activation of TAS2R significantly inhibits the LPS-induced pro-inflammatory mediators release in human blood (TNFα, IL-1β, IL-2, IL-4, IL-5, IL-10, IL-13, IL-17) [34]. In human gingival fibroblasts, LPS-induced pro-inflammatory cytokines (IL-6 and IL-8) are suppressed by TAS2R activation [13]. All of these studies suggest that TAS2R has a potential role in controlling inflammation. Pulpitis is characterized by the production of high levels of pro-inflammatory cytokines including TNFα, IL-4, IL-6, IL-8, IL-10 and CXCL8 [2, 4]. We speculate that TAS2R promotion of pulp repair is not only related to the increase in hDPSCs differentiation, but also involved in its anti-inflammatory function, implying its therapeutic potential for pulpitis in the early stage.

In the pulpitis, LPS, TNFα and IL-6 are released at high levels. Therefore, we stimulated hDPSCs to produce inflammatory microenvironment by LPS, TNFα or IL-6. We found that LPS, TNFα or IL-6 could increase the ALP, RUNX2, DMP-1 and DSPP expressions and promote odontoblastic differentiation of hDPSCs. Microarray results showed that ALP and RUNX2 were upregulated, suggesting the hDPSCs in the inflammatory pulp tissues had differentiation activity, because ALP and RUNX2 were markers of odontoblastic differentiation in hDPSCs at early stage and were highly expressed during the repair of dental pulp injuries [35, 36]. But the clinically inflammatory pulp tissues used by microarray were the samples diagnosed as irreversible pulpitis. The initial odontoblasts in the inflammatory pulp have been partially damaged, and DMP-1 was secreted by the odontoblasts [37], so the expression of DMP-1 was down-regulated in the microarray results.

To better understand the role of TAS2R in odontoblastic differentiation of hDPSCs and pulp regeneration, TAS2R10 was overexpressed in hDPSCs, and the expression markers related to odontoblastic differentiation significantly increased. We also found that when TAS2R10 was silenced, the efficiency of odontoblastic differentiation of hDPSCs was reduced (compared with LPS + ODI group), but higher than that of ODI group. We speculate that inflammation promotes odontoblastic differentiation of hDPSCs may also involve other signal pathways like TLR4, ERK, P38 MAPK, AKT, or miRNA21/STAT3 [10, 19], and inflammation activation of TAS2R10 is only one of these pathways.

TAS2R and its downstream signaling have been found in multiple extraterritorial tissues throughout the body such as digestive system [38, 39], reproductive system [40] and cardiovascular system [41]. Intracellular Ca^2+^ elevation is a downstream effect mediated by TAS2R that plays crucial roles in numerous physiological and pathological activities. Deshpande et al. [33] have reported that TAS2R is expressed in the human airway smooth muscle cells and the activation of TAS2R increases intracellular Ca^2+^ concentration, evokes airway relaxation and reduces airway hyper-responsiveness. TAS2R negatively regulates the release of thyroid-stimulating hormone through increasing Ca^2+^ in thyrocytes [42]. Previous studies have also suggested that intracellular Ca^2+^ positively regulates the odontoblastic differentiation of hDPSCs [27] and Ca^2+^ levels regulate dentin formation in vitro and in vivo [28]. This is consistent with the results of our study that activation of TAS2R10 promoted odontoblastic differentiation of hDPSCs, which was indeed regulated by Ca^2+^.

## Conclusions

In summary, our study demonstrated that TAS2R was found to be expressed in hDPSCs, and TAS2R regulated odontoblastic differentiation of hDPSCs by mediating intracellular Ca^2+^ in inflammatory microenvironment, and shed a new light on hDPSCs-based therapeutic strategies for regenerative endodontics.

## Data Availability

The authors confirm the availability of all data generated or analyzed in this manuscript.

## Abbreviations

hDPSCs: Human dental pulp stem cells
TAS2R: Bitter-taste receptors type 2
GPCR: G protein-coupled receptors
GO: Gene ontology
KEGG: Kyoto Encyclopedia of Genes and Genomes
LPS: Lipopolysaccharide
TNFα: Tumor necrosis factor α
IL-6: Interleukin 6
ODI: Odontoblastic differentiation medium
QUN: Quinine
siRNA: Small interfering RNA
qPCR: Quantitative real-time polymerase chain reaction
ALP: Alkaline phosphatase
RUNX2: Runt-related transcription factor 2
DSPP: Dentin sialophosphoprotein
DMP-1: Dentin matrix acidic phosphoprotein 1
